# An extensible six-step methodology to automatically generate fuzzy DSSs for diagnostic applications

**DOI:** 10.1186/1471-2105-14-S1-S4

**Published:** 2013-01-14

**Authors:** Antonio d'Acierno, Massimo Esposito, Giuseppe De Pietro

**Affiliations:** 1 Institute of Food Sciences - National Research Council of Italy, Via Roma 64, Avellino, Italy; 2 Institute for High Performance Computing and Networking - National Research Council of Italy, Via P. Castellino 111, Napoli, Italy

## Abstract

**Background:**

The diagnosis of many diseases can be often formulated as a decision problem; uncertainty affects these problems so that many computerized Diagnostic Decision Support Systems (in the following, DDSSs) have been developed to aid the physician in interpreting clinical data and thus to improve the quality of the whole process. Fuzzy logic, a well established attempt at the formalization and mechanization of human capabilities in reasoning and deciding with noisy information, can be profitably used. Recently, we informally proposed a general methodology to automatically build DDSSs on the top of fuzzy knowledge extracted from data.

**Methods:**

We carefully refine and formalize our methodology that includes six stages, where the first three stages work with crisp rules, whereas the last three ones are employed on fuzzy models. Its strength relies on its generality and modularity since it supports the integration of alternative techniques in each of its stages.

**Results:**

The methodology is designed and implemented in the form of a modular and portable software architecture according to a component-based approach. The architecture is deeply described and a summary inspection of the main components in terms of UML diagrams is outlined as well. A first implementation of the architecture has been then realized in Java following the object-oriented paradigm and used to instantiate a DDSS example aimed at accurately diagnosing breast masses as a proof of concept.

**Conclusions:**

The results prove the feasibility of the whole methodology implemented in terms of the architecture proposed.

## Background

Making an effective and efficient medical diagnosis is pivotal in clinical daily practice, clearly because of the impact of this singular decision making process in the eventual illness trajectory and disease management. For such a reason, the optimization of the diagnostic process in terms of number and duration of patient examinations, with corresponding accuracy, sensitivity, and specificity, is known to reduce morbidity and mortality rates, control costs and improve both doctor-patient and community-facility relationships [1].

The task of medical diagnosis, like almost any other diagnostic process, is made more complex to obtain even for a medical expert because of a web of relevant uncertainties, in the form of information incompleteness, impreciseness, fragmentariness, not fully reliability, vagueness and contradictoriness [2]. Specifically, patients may not be able to describe exactly the natural history of their disease in terms of what has happened to them or how they feel; doctors and health care practitioners may not understand or interpret exactly what they hear or observe; the accuracy of available laboratory reports, which may come with some degree of error; and the effects of treatment in an individual patient or in a group or population as a whole in terms of how diseases alter the normal functioning of the body [3]. The need to identify the most accurate medical diagnosis in a very timely manner increases dramatically in the case of mortal diseases, as both the rapid and accurate diagnosis and prompt initiation of treatment are recognized as necessary conditions to limit further complications, cut costs and reduce human suffering.

In order to improve the possibility of early and accurate diagnosis of illness, there is thus the need for the application of Diagnostic Decision Support Systems (in the following, DDSSs) in the process, because these are known to improve practitioner's performance, reduce costs and improve patient outcomes [4].

The first DDSSs proposed in literature relied on crisp models based on thresholding for solving medical classification problems. Nevertheless, they neither take into account the fuzziness of input data nor reproduce the expert decision-making process applied in a vague-laden domain such as medicine. As a matter of fact, the decision-making model every trustworthy physician has in mind to perform heuristic diagnosis is often pervaded by uncertainty and vagueness.

Recently, DDSSs based on a multi-valued logic and, in particular, on Fuzzy Logic, have been applied to medical classification problems (see, for example, [5], [6] and [7]) demonstrating their capability both to overcome the problem of managing imprecise and uncertain knowledge and offer a support for the medical decision making process. Moreover, Fuzzy Logic is quite close to natural language and allows presenting the results to clinicians in a more natural form. This makes fuzzy based DDSSs more acceptable to human users than black box systems, because both the semantic expressiveness and reasoning (by using linguistic variables and rules) are comprehensible and may be validated by human inspection.

### Crisp and fuzzy modeling

The first DDSSs were mainly based on Crisp Logic, which provides an inference morphology for drawing conclusions from existing neat and clear-cut information: specifically, new truths can be inferred from old ones. In more detail, it relies on rules, defined as conditional statements written in the following form: *if crispAntecedent then crispConsequent *where *crispAntecedent *is a crisp-logic expression made of one or more simple *predicates *linked by logical operators and depending on input *crisp variables*, and *crispConsequent *is an expression of output variables which are assigned *crisp values*. A *crisp variable C *(also named *feature*) is characterized by a set of *crisp values c *it can assume, whereas a *predicate P *classifies the values belonging to a variable into two groups or categories, i.e. values that make it either true or false, respectively. In other words, the set defined by *P(c) *is written as: {*c*|*P*(*c*)}, and is just a collection of all the values for which *P *is true. For instance, {*c*|*c *is a positive integer less than 3} is the set {1,2}.

Crisp modeling is the task of determining the parameters characterizing a rule base, classified into the followings:

• Structural parameters. Related mainly with the size of the rule base, they include the number of variables involved in the rules and the number of rules.

• Connective parameters. Related with the topology of the rule base, these parameters include antecedents, consequents, and weights of the rules.

Unlike Crisp Logic, Fuzzy Logic resembles human reasoning in its use of vague information to generate decisions [8], where vague predicates are used and values belonging to a variable cannot be classified into two groups (either true or false). In this sense, Fuzzy Logic incorporates an alternative way of reasoning, which allows modeling complex systems using a higher level of abstraction originating from knowledge and experience [9].

In more detail, in Fuzzy Logic, *a fuzzy variable F *(also named *linguistic variable*) represents a concept that is measurable in some way either objectively or subjectively and is defined by a set of *fuzzy terms T *(also named *fuzzy sets*), and by the *membership functions μ_T _*associated to these terms; fuzzy terms set a membership value from 0 to 1 to elements *u *within a predetermined range *U *(named the universe of discourse) as follows:

(1) $$T=\left\{\left(u,\mu_{T}\right)|u\in U\;and\;\mu_{T}:U\to \left[0,1\right]\right\}$$

**Figure 1:**
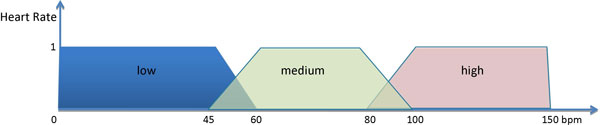
**An example of linguistic variable**.

The central notion, thus, is that truth values or membership values are indicated by a value on the range [0, 1], with 0 representing absolute false and 1 representing absolute truth. For instance, Figure 1 shows the linguistic variable *Heart Rate *made of three terms (*low, medium *and *high*), and defined in *U *= [0, 150] *bpm *(beats per minute).

The fuzzy inference morphology relies on a Fuzzy Inference System (in the following, FIS) based on *if fuzzyAntecedent then fuzzyConsequent *rules, where *fuzzyAntecedent *is a fuzzy-logic expression made of one or more simple *fuzzy expressions *linked by fuzzy operators and depending on input *fuzzy variables*, and *fuzzyConsequent *is an expression of the output variables which are assigned *fuzzy terms*.

Essentially, fuzzy reasoning is made of four steps, namely *fuzzification *of input variables, *rule evaluation, aggregation *of rule outputs and, finally, *defuzzification*. *Fuzzification *of input variables converts crisp (realvalued) inputs into fuzzy terms. For each fuzzy rule, *Rule evaluation *applies such fuzzified inputs to its antecedents, making use of a fuzzy operator in case of multiple antecedents, with the final aim of generating a single value indicating its degree of activation. Such a value is, then, used in the implication to infer the conclusion of the rule. *Aggregation *combines the membership functions of all rule consequents previously evaluated in order to generate a single fuzzy set as output. Finally, *defuzzification *determines the best representative crisp value of this aggregated output fuzzy set.

Fuzzy modeling is the task of determining the parameters of a FIS, classified into the followings [10]:

• Logical parameters. They include the shape of the membership functions, the fuzzy logic operators applied for AND, OR, implication, and aggregation operations, and the defuzzification method.

• Structural parameters. Related mainly with the size of the fuzzy system, they include the number of variables involved in the rules, the number of membership functions defined for each linguistic variable, and the number of rules.

• Connective parameters. Related with the topology of the system, these parameters include antecedents, consequents, and weights of the rules.

• Operational parameters. These parameters define the mapping between linguistic and numeric representations of the variables, so characterizing the membership functions of the linguistic variables.

The most usual, and cheapest, way for modeling medical knowledge in fuzzy-based DDSSs is asking an expert to write if-then rules. Moreover, after formalizing the expert's knowledge under the form of rules, the designer and the expert have to choose the shape and location of membership functions for all the linguistic values related to all the variables involved. This step, sure enough, requires both medical expertise and technical intervention along with great effort to identify which among the design choices are suited to the given problem. Alternatively, an emerging solution is represented by *data driven fuzzy modeling*, that is being widely adopted in different application domains to automatically generate a rule base from data, even if the interpretability is not guaranteed in many situations and redundancy can occur in the rules produced.

According to [11] a fuzzy model is interpretable when: *(i) *the fuzzy terms associated to a variable (usually indicated as fuzzy partition) are interpretable as linguistic labels, *(ii) *the rule base is as small as possible, and *(iii) *the if-part of each rule does not includes all the independent variables but only a subset of them.

### State of the art

A number of fuzzy-based DDSSs has faced up these challenges, and has attempted to address the subjects of knowledge acquisition, representation, and utilization in medical diagnosis.

In [12], a rule-base self-extraction and simplification method is proposed, devised to establish interpretable fuzzy models from numerical data. A fuzzy clustering technique associated with a fuzzy partition validity index is used to extract the initial fuzzy rule-base and find out the optimal number of fuzzy rules. To reduce the complexity of fuzzy models while keeping good model accuracy, some approximate similarity measures are presented and a parameter fine-tuning mechanism is introduced to improve the accuracy of the simplified model. Experimental results are reported with respect to different case studies, such as function approximation, dynamical system identification and mechanical property prediction for hot rolled steels. These test-cases are characterized by a relatively small number of input-space variables. No experimental test has been reported for problems characterized by a high number of input-space variables.

In [13], an evolving hierarchical fuzzy system based on probabilistic incremental program evolution is presented. The use of hierarchical fuzzy systems allows to limit both the number of rules and the number of fuzzy operations with respect to single level systems. Worthy results are described for case studies concerning non linear system identification, such as Makey-Glass chaotic time series prediction, and the Iris and Wine classification.

In [14] a data-driven innovative approach is presented for generating a fuzzy rule based decision support system for the diagnosis of coronary artery disease. The implemented methodology relies on four stages.

In the first stage, a decision tree is induced from the dataset, while in the second stage, a set of rules is extracted from it. This set of rules is in *Disjunctive Normal Form *(DNF) and involves crisp variables modeling neat and clear-cut quantities. It can be used as a whole to classify new incoming data coherently with the knowledge embedded into the initial dataset. In the third stage, the crisp model is fuzzified, i.e., the crisp rules are transformed into fuzzy ones, using a fuzzy membership function instead of the crisp one and definitions of S and T norms. Finally, in the fourth stage, the parameters entering the fuzzy model are adapted using a global optimization technique.

In [15] a generic methodology is presented for the automated generation of fuzzy models. The methodology is realized in three stages. Initially, a crisp model is created whereas, in the second stage it is transformed into a fuzzy one. In the third stage, all parameters entering the fuzzy model are optimized. A specific realization of this methodology is implemented, using decision trees for the creation of the crisp model, the sigmoid function, the min-max operators and the maximum defuzzifier for the transformation of the crisp model into a fuzzy one, and four different optimization strategies, including global and local optimization techniques, as well as hybrid approaches.

In [16] a generic approach to the design of interpretable data-driven fuzzy models, which can be used in the construction of DDSSs, is proposed. The approach addresses several design steps, including fuzzy partitioning, rule learning, variable selection and rule base simplification. The fuzzy partitioning step consists in generating a collection of fuzzy partitions of various sizes from two to a user-defined maximum value, based upon indices or an objective function. The rule learning step includes two categories of methods, namely region based methods and prototype based ones. The rule base simplification merges some rules into a more generic incomplete rule, where some variables (one or more) appear in some rules. The variable selection determines the number of terms for a given variable necessary to get a good rule base, in terms of trade-off between its complexity and accuracy, measured by performance indexes.

### The contribution of the work

Recently, we informally introduced [17,18] a methodology to design and develop a fuzzy-based DDSS for medical classification problems by extracting fuzzy knowledge from data. In this work, we first propose a formalization of a refined and assessed version of such a methodology, which essentially specifies a flow of stages needed to develop a fuzzy-based DDSS as well as the characteristics of the input and output produced and consumed in the different stages. As a result, it formalizes the role assumed by each stage in terms of its interface, but it does not provide any indication about how the single activities have to be done in the form of strategies to be adopted or algorithms to be applied, especially because this choice is strictly linked to the specific application domain. This issue is further motivated by the fact that the methodology, whose strength relies on its generality and modularity, has been thought as a basis for the development process of fuzzy-based DDSSs by supporting the integration of alternative techniques in each of its stages. Both the generality and flexibility make it applicable to almost any medical classification domain and, also, enable the possibility to test the efficiency of different methods in order to detect their best integration with respect to specific classes of problems.

**Figure 2:**
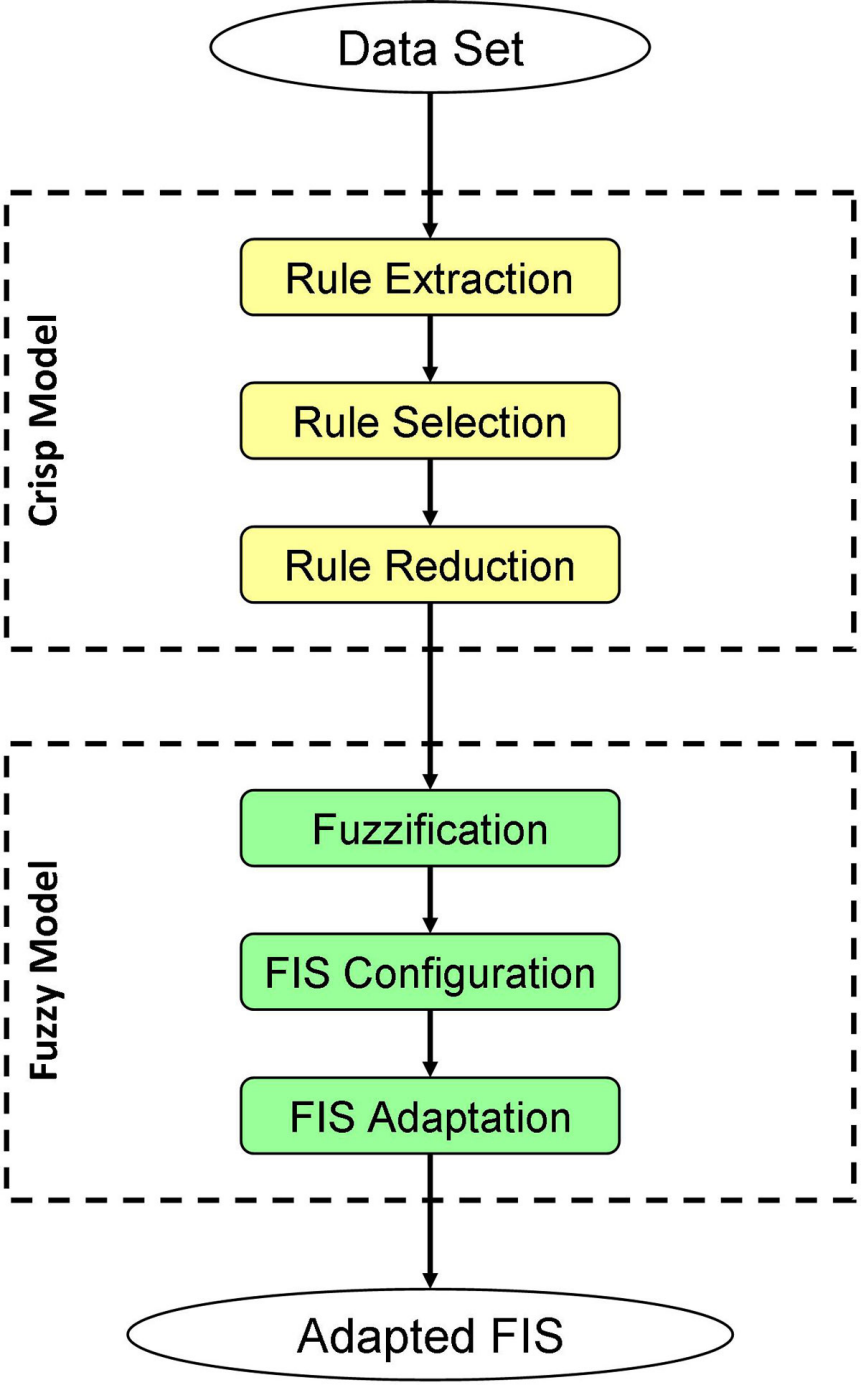
**Our six-step methodology in terms of activity diagram**.

In more detail, the assessed version of the methodology (Figure 2) includes six stages: *(i) *extraction of crisp rules, *(ii) *selection of a significant partition from the extracted rule set, *(iii) *reduction of the selected rule set, *(iv) *creation of fuzzy rules, *(v) *generation of the whole fuzzy inference system and *(vi) *its optimization. In the first three stages a set of crisp rules is initially created and, then, appropriately elaborated in order to be compliant with some characteristics, which are strongly necessary for this methodology to make the fuzzification feasible. In this work, such characteristics are formulated with the definition of *fuzzifiability*. Successively, the last three stages are in charge of *(i) *transforming the (*fuzzifiable*) crisp rules into the corresponding fuzzy versions, i.e. in terms of connective and structural parameters, *(ii) *defining the most appropriate logical parameters to be used in the fuzzy inference system and *(iii) *optimizing all the operational parameters composing the fuzzy rules and, if required, also the relative relevance of each of them, specified in the form of a weight.

The methodology has been realized in the form of a modular and portable architecture according to a *Component-Based Software Development *(in the following, *CBSD*), with the aim of defining a collection of components customizable or extensible by existing available solutions that are compatible to the original placeholders.

The architecture has been developed in Java according to the object-oriented paradigm in order to create a truly portable DDSS, solving the problem of having parts of it implemented for different platforms. The resulting architecture can be considered as well-suited for almost any medical domain where the real world is simulated in a broad sense and a diagnosis in terms of classification is required.

As a proof of concept, such an architecture has been used to instantiate a DDSS example aimed at accurately diagnosing breast masses starting from the widely used *Wisconsin Breast Cancer Dataset *(in the following, *WBCD*) to evaluate the feasibility of the methodology.

The manuscript is structured as follows: in *Methods*, the proposed methodology is formally described and the choices and techniques which are identified for the specific realization are analyzed. Furthermore, the design approach used for the development of the proposed architecture is introduced. In *Results*, the architecture designed is diffusely described and a summary inspection of the main components is reported as well. Moreover, the proof of concept DDSS for validating the methodology is introduced and its results are discussed. Finally, conclusions and on-going activities are outlined in the last section as closure to the paper.

## Methods

Our methodology includes six stages, where the first three stages work with crisp models, whereas the last three ones are employed on fuzzy models (Figure 2).

### Rule extraction

The *Rule Extraction *stage is essentially devised to the extraction, from a specific input dataset, of a collection of *if-then *rules constituting the crisp rule base (in the following, *CRB*), specifically represented in a *weighted Disjunctive Normal Form (wDNF)*. We work with this representation because of its high degree of compactness and knowledge synthesis. The *CRB*, thus, is a disjunctive system of crisp rules, where at most one rule must be satisfied by an item of the initial dataset, i.e. the rules are linked by mutually exclusive or connectives. More formally, each crisp rule $r_{ij}^{c}$ (note that the superscript *c *is used to label a rule as crisp) in the *CRB *denotes the *i^th ^*rule which predicts the *j^th ^*class, with *j *= 1... *M *and *i *= 1... *N_j_*, where *M *is the total number of classes and *N_j _*is the total number of rules that predict the *j^th ^*class, respectively. As a result, in general, there could be one or more than one rule for each class predicted, while each class is likely to be covered by at least one rule in the *CRB*.

The structure of each rule $r_{ij}^{c}$ is composed of a conjunction of antecedent predicates (which can be evaluated to be either true or false), based on the set of features $X^{c}=\left\{x_{l}^{c}\right\}$, with *l *= 1... *L *(where *L *is the number of features of the initial dataset), and one consequent term indicating the specific class predicted. More in detail, for the *i^th ^*rule which predicts the *j^th ^*class, given the sets of crisp predicates $P_{ij}^{c}=\left\{p_{ijk}^{c}\right\}$, with *k *= 1... *K_ij _*(where *K_ij _*is the total number of predicates for *i^th ^*rule which predicts the *j^th ^*class), and let $y_{j}^{c}$ be the consequent crisp term representing the predicted *j^th ^*class, its formulation is defined as:

(2) $$r_{ij}^{c}\;:\;p_{ij1}^{c}\left(X^{c}\right)\wedge \ldots .\wedge p_{ijK_{ij}}^{c}\left(X^{c}\right)\to y_{j}^{c}$$

with each crisp predicate expressed as:

(3) $$p_{ijk}^{c}\left(X^{c}\right)\equiv \left(x_{ijk}^{c}op^{c}v_{ijk}^{c}\right)$$

where $x_{ijk}^{c}$ is the specific feature selected from the set *X^c^*, $op_{ijk}^{c}$ is a comparison operator selected from the sets {=, ≠} and {<, >, ≤, ≥} in the cases of categorical and numerical features, respectively, and $v_{ijk}^{c}$ represents a categorical value or a crisp numerical threshold.

This DNF is labeled as *weighted *since each rule is associated with a degree of relevance, such as its coverage or accuracy, with respect to its predicted class depending on the domain of application and the specific dataset concerning this domain.

Different solutions can be adopted to extract rules, ranging from purely logical approaches to statistically-based ones or relying on artificial neural networks, genetic algorithms and on non-connectionist machine learning (e.g. decision trees) [19]. Independently from the specific method used, it is relevant to point out that it is possible to extract rules able to correctly classify an item in the dataset from its known features, i.e. every item in the input dataset is covered by exactly one rule in the *CRB*, but without avoiding the possibility of overfitting the input dataset which can be characterized by a degree of uncertainty. This uncertainty may arise from two different sources. The first is mis-measurement, i.e., for a variety of reasons, the value of a feature or class may be incorrectly measured. The second source of uncertainty is the occurrence of extraneous factors not recorded, but affecting the results so that the class of an item in the dataset cannot be determined wholly from its recorded features. The resulting *CRB *extracted in these situations tends to be very large and many rules reflect particular items in the training dataset which are very unlikely to occur in further examples, i.e. they cover a very small part of the input space, are matched only by a few examples, lack generality and can become counter-productive. This issue represents the motivation for the second stage, i.e. *Selection*.

### Selection

The *Selection *stage is in charge of determining the sufficient number of rules, as are necessary to get a good *CRB *with respect to the specific dataset concerning the domain under observation, where a good *CRB *represents a reasonable trade-off between complexity, determined by the number of rules, and accuracy, measured by appropriate performance indexes. The selection is done with the idea of granting two main factors emerged as primary determinants of interpretability. First, the number of rules should be small so involving that a full set of complete rules should be avoided since it can quickly lead to a combinatorial explosion when the number of features rises [16]. The second condition is strictly linked to the first one and is specific to complex systems with a large number of features: rules must not systematically include all the features, but only the important ones in the context of the rule, so generating the often called incomplete rules [16].

Different methods can be applied to perform the rule selection and, thus, implicitly also the variable selection, each of them exploiting ad-hoc heuristics guided by user-defined indicators (such as the final number of rules to be chosen, from a minimum of one for each predicted class to a maximum which can be properly indicated by the user) and suitable with respect to the different applications and their specific requirements. For example, the choice of the good *CRB *can be piloted by a longest-match criterion, i.e. depending on the longest left-hand-side (LHS) of the rules that match an item of the dataset. Its rationale is based on the conclusion that longer antecedent part will contain more accurate and richer information for the final classification than the shorter ones. Differently, a most confident selection could be adopted, by identifying the rules with the highest confidence as the best one, where the longer rule is chosen in case of a tie. The rationale of this criterion is based on the assumption that the testing dataset will share the same characteristics as the training dataset used in the *Rule Extraction*. So if a rule has a high confidence in the training data, then this rule will also show a high confidence in the testing data, which means the class predicted by that rule will be most likely to occur next.

Summarizing, this stage has a double relevance in terms of the functionalities offered: first, it performs the rule selection to achieve a smaller number of more general rules with the idea that it may have greater predictive accuracy on unseen data, at the expense of no longer correctly classifying some of the items in the original training dataset; then, it implicitly carries out also a feature selection leading to an incomplete rule base which takes into consideration only those features that are really required since included into the significant rules previously selected.

It is worth noting that the elimination of features in order to obtain incomplete rules could be undertaken at the extraction level since the *Rule Extraction *step can remove features from the whole rule, for example using statistical indexes. Differently, this stage is intended to select only a subset of the features instead of the whole set of candidate ones for other reasons: *i) *it can be cheaper to measure only a reduced set of features; *ii) *prediction accuracy may be improved through exclusion of poorly significant features; *iii) *the final DDSS to be built might be simpler and potentially faster when fewer input variables are used; *iv) *knowing which features are more relevant can give insight into the nature of the classification problem and allow a better understanding of the final DDSS. At this point, in the context of the same rule *r_ij _*given as output after the *Selection*, with respect to numerical features, different predicates can contain the same feature selected from the set *X^c^*, i.e. $x_{ijl}^{c}=x_{ijk}^{c}$ with *l, k *∈ [1, ..., *K_ij_*] and *l *≠ *k*. This consideration represents the motivation for the third stage, *Reduction*,

### Reduction

The *Reduction *stage is in charge of simplifying the structure of each crisp rule in order to make it compliant with some characteristics, which are strongly necessary for this methodology to make the fuzzification feasible. In this work, such characteristics are formulated with the definition of *fuzzifiability*. A crisp rule is defined as *fuzzifiable*, if and only if each of its numerical features appears in one or at most two predicates in its antecedent part, according to one of the following forms:

(4) $$p_{I}^{c}\left(x^{c}\right)\equiv \left(x^{c}<v^{c}\right)$$

(5) $$p_{II}^{c}\left(x^{c}\right)\equiv \left(x^{c}>v^{c}\right)$$

(6) $$p_{III}^{c}\left(x^{c}\right)\equiv \left(x^{c}<v_{1}^{c}\wedge x^{c}>v_{2}^{c}\;with\;v_{1}^{c}>v_{2}^{c}\right)$$

This stage thus puts into effect a simplification procedure, that iteratively searches, in the context of each rule, each couple of predicates involving the same feature, using comparable operators and needed of being made compatible with the fuzzifiability. It is important to point out the meaning of comparable operators. Two comparison operators are intended as comparable in this procedure if and only if they appear in one of the situations reported in 7, independently of their order:

(7) $$comp\equiv \left(op_{1}^{c}{=}^{\prime }\geq^{\prime }\wedge op_{2}^{c}{=}^{\prime }{>}^{\prime }\right)\vee \left(op_{1}^{c}{=}^{\prime }\leq^{\prime }\wedge op_{2}^{c}{=}^{\prime }{<}^{\prime }\right)$$

When a couple of candidate predicates is detected, since in the rule under evaluation they are connected by conjunctions (remember that this is due to the fact that the *CRB *is in DNF), they can be reduced as follows:

(8) $$\left(x^{c}op_{1}^{c}v_{1}^{c}\right)\wedge \left(x^{c}op_{2}^{c}v_{2}^{c}\right)\to x^{c}>max\left(v_{1}^{c},v_{2}^{c}\right)\;if\;\left(op_{1}^{c}\in \left\{\geq ,>\right\}\wedge op_{2}^{c}\in \left\{\geq ,>\right\}\right)$$

(9) $$\left(x^{c}op_{1}^{c}v_{1}^{c}\right)\wedge \left(x^{c}op_{2}^{c}v_{2}^{c}\right)\to x^{c}<min\left(v_{1}^{c},v_{2}^{c}\right)\;if\;\left(op_{1}^{c}\in \left\{\leq ,<\right\}\wedge op_{2}^{c}\in \left\{\leq ,<\right\}\right)$$

After applying this simplification procedure to every couple of candidate predicates, each resulting predicate will be formulated according to one of the forms defined in equations 4-6. A clarifying note has to be reported about the categorical features. As they assume mutual exclusive values and, in each rule, the antecedent predicates are admitted to be connected only by means of conjunction operators, it is not possible (since meaningless) at all that two different predicates might contain the same feature assuming different values in the context of the same rule. Thus, the *Reduction *does not involve the categorical features in its simplification procedure.

### Fuzzification

After the first three stages, a crisp model made of rules based on clear-cut boundaries has been generated in accordance with the fuzzifiability property. Successively, starting from the crisp model produced, the generation of the fuzzy model begins with the fourth stage, named *Fuzzification*.

In more detail, in order to create fuzzy variables and terms, in this stage, crisp rules are translated into a corresponding fuzzy version, where every feature contained in the crisp rules is associated to a linguistic variable. It is relevant to mention that only features determined relevant to the classification by means of the previous stages are fuzzified. Of course, also the predicted class is associated to a linguistic variable. Successively, each linguistic variable is further characterized by a set of terms subjectively describing it. With respect to this issue, the degree of detail to be used in partitioning the universe of discourse of each variable, i.e. number of linguistic terms to be referred to it, has been chosen in accordance with the crisp model. Summarizing, the fuzzy rule base (in the following, *FRB*) achieved in this stage continues to be a disjunctive system of rules. Nevertheless, differently from the crisp case, where exactly one rule must be satisfied, the fuzzy rules are linked by simple *OR *connectives, where at least one rule must be satisfied, i.e. one or more rules may be weakly or strongly activated simultaneously.

Each fuzzy rule $r_{ij}^{f}$ (note that the superscript *f *is used to denote a rule as fuzzy) in the *FRB *denotes the *i^th ^*rule which predicts the *j^th ^*class, with *j *= 1... *M *and *i *= 1... *N_j_*, where *M *is the total number of classes and *N_j _*is the total number of fuzzy rules that predict the *j^th ^*class, respectively. The structure of each fuzzy rule $r_{ij}^{f}$ is composed of a conjunction of antecedent fuzzy predicates, based on the set of linguistic variables $X^{f}=\left\{x_{u}^{f}\right\}$, with *u *= 1, ..., *U_c_*, (where *U_c _*is the number of features effectively used in the crisp model), and one consequent fuzzy variable indicating the specific class predicted. More formally, for *i^th ^*rule which predicts the *j^th ^*class, given the sets of fuzzy predicates $p_{ij}^{f}=\left\{p_{ijk}^{f}\right\}$, with *k *= 1... *K_ij _*(where *K_ij _*is the total number of predicates for *i^th ^*rule which predicts the *j^th ^*class), and let $y_{j}^{f}$ be the consequent linguistic variable representing the predicted *j^th ^*class, its formulation is defined as follows:

(10) $$r_{ij}^{f}\;:\;p_{ij1}^{f}\left(X^{f}\right)\wedge \ldots \wedge p_{ijK}^{f}\left(X^{f}\right)\to y_{j}^{f}$$

with each fuzzy predicate expressed as:

(11) $$p_{ijk}^{f}\left(X^{f}\right)\equiv \left(x_{ijk}^{f}\;is\;v_{ijk}^{f}\right)$$

where $v_{ijk}^{f}$ represents a (*fuzzified*) numerical interval in the case $x_{ijk}^{f}$ is a fuzzy variable associated to a numerical feature or a (*fuzzified*) categorical value in the case $x_{ijk}^{f}$ is fuzzy variable associated to a categorical feature. Finally, also the *FRB *is weighted since each fuzzy rule is associated with the same degree of relevance pertaining the crisp rule it has been generated from.

Different methods can be applied to fuzzify crisp rules [14], [15], [20], [21], ranging from solutions which, on the one hand, exploit the symbolic structure of the crisp rules to generate fuzzy variables and terms to be inserted into the predicates of the corresponding fuzzy rules and, on the other hand, soften the sharp crisp thresholds to minimise continuous terms close to the decision boundaries from misclassification, to approaches where the crisp rule structure is only used to define fuzzy variables and terms and the sharp thresholds are not taken into account at all into the fuzzification process.

### FIS configuration

**Figure 3:**
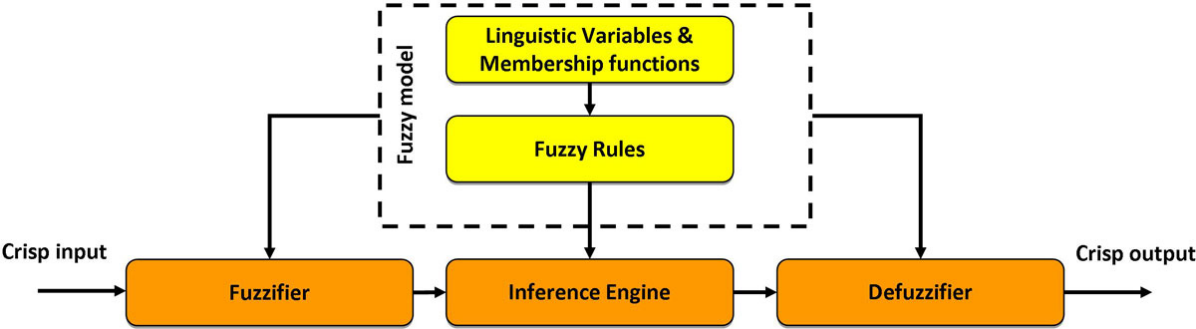
**A Fuzzy Inference System**.

At this point, after generating the fuzzy model in terms of rules, linguistic variables and terms, the overall Fuzzy Inference System underpinning the DDSS has to be generated in the fifth stage, named *FIS Configuration*, depending on the domain of application and its specific requirements. As depicted in Figure 3, a FIS is a system aimed at solving a typically complex and nonlinear problem by utilizing fuzzy logic methodologies. Its basic structure includes four main components, namely a *Fuzzifier *(which translates real-valued inputs into fuzzy values), an *Inference Engine *(that applies a fuzzy reasoning mechanism to obtain a fuzzy output), a *Defuzzifier *(to translate this latter output into a crisp value), and a *Fuzzy model *(containing fuzzy rules, linguistic variables and membership functions).

Connective and structural parameters of the FIS (to be generated) have been thus defined in the previous stage. Differently, in this stage, the most appropriate logical parameters to be used in the FIS have to be determined by the designer based on experience and depending on the domain characteristics. Typical choices for the reasoning mechanism are Mamdani-type, Takagi-Sugeno-Kang (TKS)-type, and Singleton-type. Common fuzzy operators are min, max, product, probabilistic sum, and bounded sum. The most common membership functions are triangular, trapezoidal, gaussian and bell-shaped. For defuzzification several methods have been proposed with the center of area (COA) and the mean of maxima (MOM) methods being the most popular. Moreover, depending on the typology of reasoning mechanism desired, different Inference Engines can be used, for instance, for supporting the rule chaining [22] or operating in accordance with an If-Then-Else rule structure [23].

### FIS adaptation

After determining the most appropriate logical parameters, in order to complete the generation of the FIS underpinning the DDSS, the operational parameters have to be identified in the last stage, named *FIS Adaptation*, in terms of parameters characterizing shape and location of membership functions for all the terms related to all the linguistic variables involved in the *FRB*.

For what concerns linguistic variables linked to categorical features, the determination of shape and location of their membership functions is very simple. Since a categorical feature is one that has two or more categories, but there is no intrinsic ordering to them, they can be modeled as independent singular singletons. Referring to linguistic variables linked to numeric features, a tuning process considering the whole FRB obtained has to be used a posteriori to adjust the membership function parameters. The classic way to refine the membership functions is to adjust through slight modifications their parameters in order to find the local or global minimum of a mono/multi objective function *F(x) *opportunely defined, which takes into account specific indexes modeling at least three characteristics [24] a DDSS should possess.

First, the performance of a DDSS in performing a diagnosis can be evaluated with reference to the correct classification rate (*CR*), even if the system should jointly provide also a numerical value (the *confidence *χ) indicating its confident in the outcome produced. Furthermore, a DDSS should provide the physicians with the possibility of deeply understanding how this outcome has been generated (*interpretability*), in order to increase its trustworthiness and not to appear as a black box that produces unintelligible outputs. It is worth noting that these characteristics can often result conflicting.

This tuning process can be implemented using parameter adjustment algorithms, such as Neural Networks algorithms, and in most cases, the gradient of a cost function with respect to each adjustable parameter can be calculated and the parameters can be updated accordingly. There are also some derivative-free optimization such as Genetic Algorithms and Random Search Methods. As a concluding remark, it is worth noting that in this stage not only the operational parameters can be adapted, but also the weights of each rule in the FRB, which have been previously extracted in the first stage and associated to the fuzzy rules in the fourth stage, can be refined. Such a way, it is possible to induce a better cooperation among rules and to more accurately modulate the firing strength of a rule in the process of determining the output class.

### Implementing the methodology

The proposed methodology has been realized in the form of a modular and portable architecture according to a *CBSD *approach, with the aim of defining a collection of replaceable (and reusable) components characterized by a functional cohesion (i.e. the single component performs a well-defined set of functions) and a low degree of coupling in terms of composition and interaction between them.

The choice of a *CBSD *approach is based on the idea that each single component can be not only implemented from scratch but, in particular, also customized or extended by existing available solutions that are compatible to the original placeholders. Such a way, the *CBSD *can significantly reduce development effort and time-to-market, and improve maintainability, reliability and overall quality of final architecture designed. The architecture is developed in Java according to the object-oriented paradigm in order to create a truly portable DDSS, solving the problem of having parts of it implemented for different platforms.

In the following section, the architecture designed will be diffusely described and a summary inspection of the main components in terms of UML class diagrams will be reported as well.

## Results

### The proposed architecture

**Figure 4:**
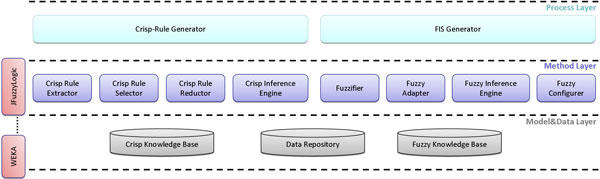
**The proposed three-layer architecture**.

The CBSD approach has generated an extensible and layered architecture. An extensible architecture has been necessary because the proposed methodology is intended to support the realization of DDSSs according to both general-purpose and special-purpose application needs. Special-purpose requirements need to be incorporated depending on specific medical scenarios, whereas, general-purpose mechanisms will be common across all applications. Moreover, it has been conceived as layered since, such a way, it can support design based on increasing levels of abstraction, thereby partitioning the overall design problem into several sub-problems. Plus, it supports enhancement and reuse since, assuming that the interfaces between the layers do not change, it allows for changes to occur within the layer in relative isolation without impacting the other layers. This improves the scalability of the architecture as well as quality and testability. Thus, such an architecture can lead to standard interfaces for each layer and its components, so that layer implementations can be re-used across different DDSSs. The system architecture adopted is shown in Figure 4.

The architecture provides for three different layers: a *Process Layer*, which coordinates the activity flow foreseen by the methodology in terms of two loosely coupled sub-processes, a *Method Layer *which handles highly cohesive and well-defined operations (from a functional perspective) to be done in each sub-process, and a *Model&Data Layer *which manages the data structures used to read and store crisp and fuzzy knowledge bases as well as data repositories. Moreover, in accordance with the CBSD approach, the architecture highlights a transversal layer, shared between the *Method Layer *and the *Model&Data Layer*, which reflects the idea that each single component at these two layers can be implemented not only from scratch but also by re-using or customizing existing available solutions, such as data mining software packages (e. g. WEKA [25]) or fuzzy logic libraries (e. g. jFuzzyLogic [26] or XFuzzy [27]).

The definition of the components for the process layer has been guided by a top-down approach, where the methodology, which can be seen as a high level business process, has been divided into two smaller, more manageable and loosely coupled sub-processes. The need for a process modularization has been pushed by different motivations. First, the proposed methodology is large and complex, and it becomes very difficult to navigate, understand, debug issues and track changes in its implementation. Moreover, referring to the stages of the methodology, the clear and well-defined separation between the crisp and fuzzy domains suggests a natural strategy of modularization from a logical and functional perspective. As a result, the methodology has been modularized in two sub-processes, one for each domain, so as to contextually balance the need of performance and manageability versus the need of reuse as well. The first sub-process, handled by the *Crisp Rule Generator*, is devised to generate a set of fuzzifiable crisp rules starting from an input dataset, whereas the second one, managed by the *FIS Generator*, is thought to produce a whole FIS starting from the output of the first sub-process. Both these components assume the role of coordinators with respect to the activity flow foreseen by each single sub-process. They coordinate all the components of the method layer by activating their functionalities, examining and validating their results, and continuing the handling of the respective sub-process accordingly.

The *Method Layer *contains the building block components for implementing each single task foreseen in both the crisp and fuzzy sub-processes. These components are implemented on top of the *Model&Data Layer *in terms of a collection of modules which accesses the respective knowledge bases and data repositories, elaborates such an information and stores the results into the knowledge bases again. Moreover, all these components can be opportunely specialized in order to support different criteria, also by wrapping or utilizing existing tools.

**Figure 5:**
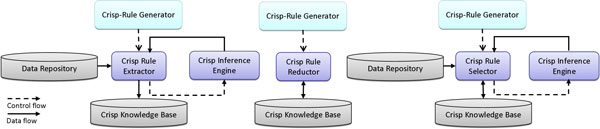
**The stages coordinated by the Crisp-Rule Generator**.

More in detail, the *Crisp Rule Extractor *(Figure 5, left) is the component in charge of first extracting a collection of crisp rules from a specific *Data Repository *and then storing it into the *Crisp Knowledge Base*. The extraction procedure can involve the strict collaboration with a *Crisp Inference Engine*, which is a component responsible of evaluating the firing of a rule with respect to a specific input data item.

The *Crisp Rule Selector *(Figure 5, right) is responsible of getting the extracted rules from the *Crisp Knowledge Base*, determining the best *n *rules (*n *is a user-defined parameter) to be successively used with respect to a specific criterion and finally storing such a new rule set into the *Crisp Knowledge Base again*. Since the criterion applied for the selection might require the identification of the set of rules with the highest confidence, such a component can interact with the *Crisp Inference Engine *to evaluate how each rule works on the dataset gathered from the *Data Repository*.

Last, the *Crisp Reductor *(Figure 5, center) is devised to get the selected rules from the *Crisp Knowledge Base*, simplify their structure in order to grant the fuzzifiability and finally store the reduced rules into the *Crisp Knowledge Base *again.

The components of the method layer described until this point cooperate among them to model the first sub-process, and, thus, they are all coordinated by the *Crisp Rule Generator*. The remaining components belonging to the method layer act together for bringing in realization the second sub-process, and, thus, they are all coordinated by the *FIS Generator*.

**Figure 6:**
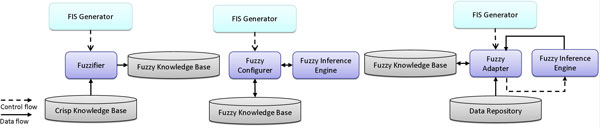
**The stages coordinated by the FIS Generator**.

More precisely, the *Fuzzifier *is designed to get the reduced crisp rules as well as the description of each single feature involved in them (e.g. in terms of range of admissible values) from the *Crisp Knowledge Base*, fuzzify them into fuzzy rules, linguistic variables and terms and store the results into the *Fuzzy Knowledge Base*, so as to actually realize the fourth stage of the methodology (see Figure 6, left). In detail, such a component makes in practice the specific fuzzification criterion described at the end of the Methods section.

Referring to the fifth stage of the methodology, the component involved is the *Fuzzy Configurer *(see Figure 6, center), which is responsible of setting the most appropriate logical parameters to be used for the construction of the final FIS, opportunely determined by the designer based on experience and depending on the domain characteristics. Moreover, depending on the typology of reasoning mechanism desired, the specific *Fuzzy Inference Engine*, aimed at performing a fuzzy inference to obtain a fuzzy output, is also configured and instantiated.

The component engaged for the realization of the last stage of the methodology is the *Fuzzy Adapter *(Figure 6, right), which is mainly devised to first get the partially defined FIS from the *Fuzzy Knowledge Base*, successively tune its logical parameters, in terms of parameters characterizing shape and location of membership functions, and rule weights, and finally store the completely defined FIS into the *Fuzzy Knowledge Base *again. Such a component strictly interacts with the *Fuzzy Inference Engine *since every optimization strategy requires the evaluation about how each fuzzy rule works on the dataset gathered from the *Data Repository*.

Finally, the *Model&Data Layer *is responsible for the management of the structures for inserting and gathering information, respectively into and from both the Crisp and Fuzzy Knowledge Bases and, in addition, for accessing the disk-based data structures used by the data repositories. In particular, with respect to the data format in the repositories, comma-separated values (CSV) are used to store tabular data (numbers and text) in a plain-text form. In particular, the first row contains the attribute names (separated by commas) followed by each data row with attribute values listed in the same order (also separated by commas). This choice is due to the fact that many data repositories or spreadsheet applications save or export data into flat files in this format.

**Figure 7:**
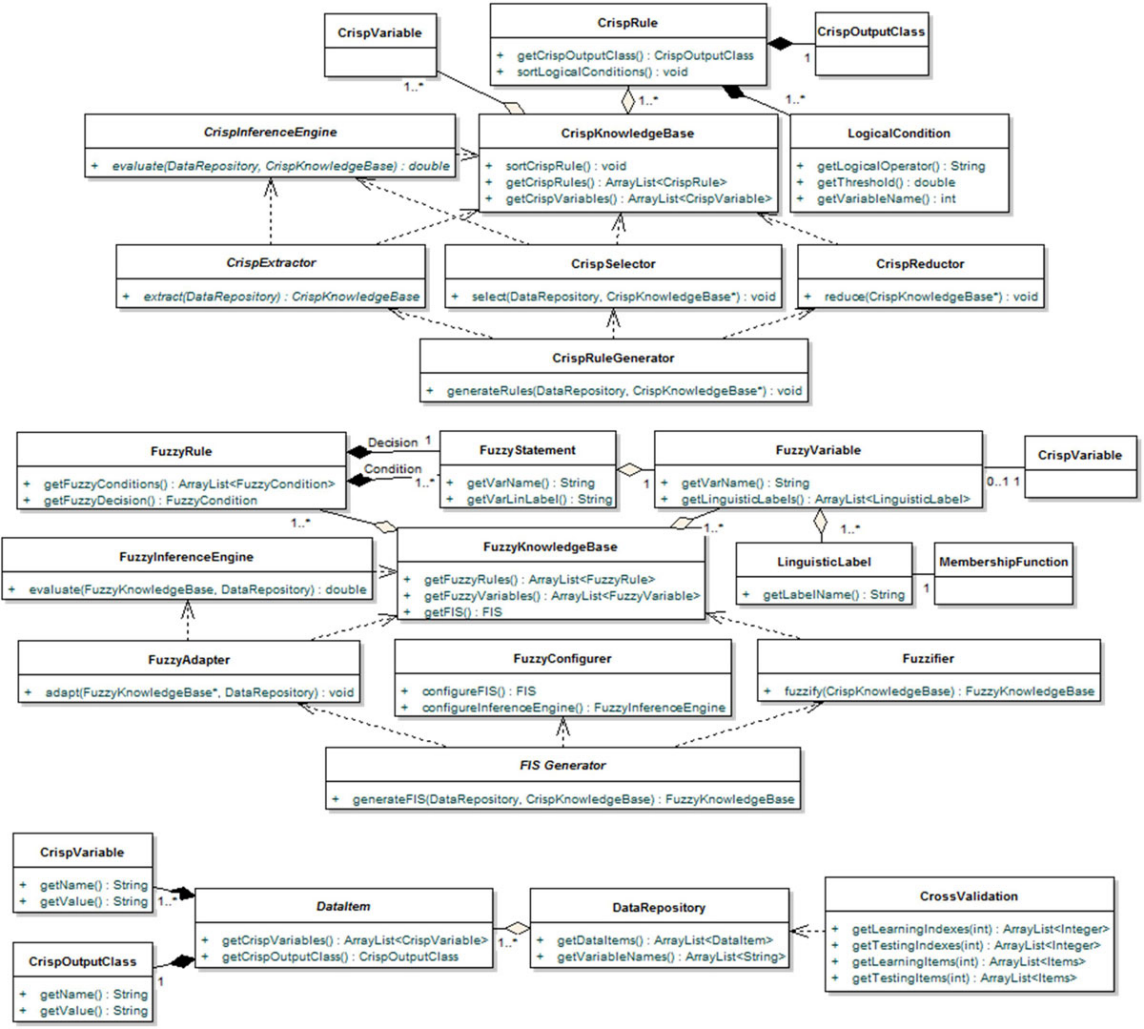
**The UML class diagrams**.

The design of this architecture in terms of software classes has been depicted as UML class diagrams, where each class has been summarily outlined below in terms of only the most significant public operations defined, with respect to the three different modules. In order to better facilitate the reading of the diagrams, note that a solid line models a structural association between two classes, whereas a broken line indicates a functional dependency between them. The first diagram includes the main classes devised for implementing the architectural components, across the three layers, which operate in the crisp domain and are responsible of realizing the first three stage of the methodology (Figure 7, top). The second one includes the main classes realizing the components which operate in the fuzzy domain and are in charge of implementing the last three stage of the methodology (Figure 7, middle). The last one includes the main classes realized for the management of the data repositories (Figure 7, bottom). These classes also offer facilities for automatically partitioning the dataset into learning and testing sets, in order to support the *n*-fold cross validation method.

### Proof of concept: a DDSS for diagnosing breast masses

The architecture has been used to instantiate a DDSS example aimed at accurately diagnosing breast masses starting from the widely used *Wisconsin Breast Cancer Dataset *(in the following, *WBCD*) to evaluate the feasibility of the methodology.

This dataset was computed from fine needle aspiration (FNA) of a breast mass through image processing and was collected at the University of Wisconsin; it can be obtained from UCI (University of California at Irvine) machine learning repository. The samples contain features describing characteristics of the cell nuclei present in the image. The version of WBCD used consists of 10 features obtained from FNA, namely radius, texture, perimeter, area, smoothness, compactness, concavity, concave points, symmetry, fractal dimension. Each feature is represented with 3 values, namely the mean, standard error and the *worst *or largest (mean of the three largest values), but only the mean value was taken into account. The two outputs are benign and malignant. All the instances were properly recorded without any missing value. The diagnosis class is distributed with 357 benign samples and 212 malignant samples.

The architecture was instantiated for creating the DDSS as described in the following. Preliminary, it is worth noting that the tenfold cross validation method was used for the assessment of such a DDSS and the classification rate was chosen as metric to evaluate the goodness of the final results achieved. Such a way, the whole methodology was tested for its validation, since the goodness of the final results was considered as proof of feasible and efficient integration of different methods according to its activity flow in order to obtain fuzzy-based DDSSs. As a result, the DDSS is described below with respect to each stage of the methodology, by reporting the partial results calculated only for a fold for the sake of brevity.

**Figure 8:**
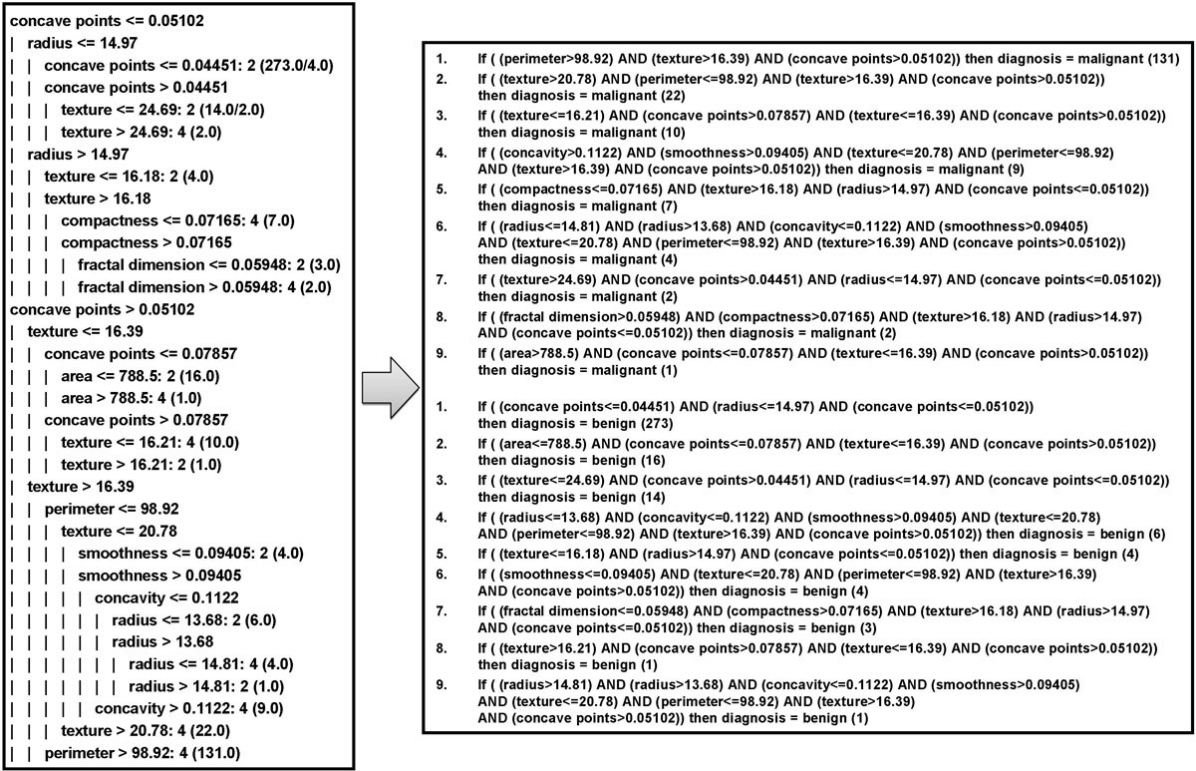
**The rules extracted**.

Crisp rules were extracted as equally weighted from a J48 decision tree, induced by WBCD. Such a method was wrapped on the top of its WEKA implementation. Figure 8 outlines the resulting decision tree and the corresponding set of rules, grouped according to the diagnosis class, i.e. malignant and benign, respectively and ordered according to their coverage, shown in brackets in Figure 8, indicating the number of correctly classified instances.

**Figure 9:**
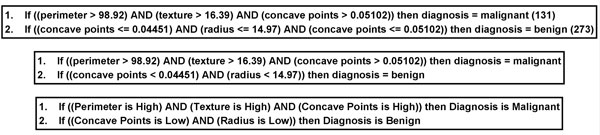
**The rules selected (top), reduced (middle), and fuzzified (bottom)**.

Successively, a simplification method based on the most confident selection with respect to the rule coverage was adopted, where the number of rules to be generated was fixed to 2, i.e. one for each output class. The selected rules (see Figure 9, top), were opportunely reduced by involving, in particular, only the rule 2 (see Figure 9, middle).

With respect to the *Fuzzification *stage, a method here proposed, based on a determinant of interpretability, was applied. In more detail, in the case of numeric features, a collection of at most three partitions is generated for the universe of discourse of each linguistic variable, by considering one crisp rule at a time and translating its predicates involving numeric features from their crisp forms to the corresponding fuzzy representations:

(12) $$p_{I}^{c}\left(x^{c}\right)\equiv \left(x^{c}<v^{c}\right)\to p_{I}^{f}\equiv \left(x^{f}\;is\;t_{I}^{f}\right)$$

(13) $$p_{II}^{c}\left(x^{c}\right)\equiv \left(x^{c}<v_{1}^{c}\wedge x^{c}>v_{2}^{c}\right)\to p_{II}^{f}\equiv \left(x^{f}\;is\;t_{II}^{f}\right)$$

(14) $$p_{III}^{c}\left(x^{c}\right)\equiv \left(x^{c}>v^{c}\right)\to p_{III}^{f}\equiv \left(x^{f}\;is\;t_{III}^{f}\right)$$

where $t_{I}^{f},\;t_{II}^{f}$ and $t_{III}^{f}$ correspond to linguistic terms (e.g. low, medium and high) linearly ordered and with boundary regions between successive terms. It is relevant to highlight that the semantics associated to each linguistic term obtained in such a way is strictly application-dependent and is meaningful only in the context of the rule for which it has been defined. As a result, this method defines as many terms for each linguistic variable as the different crisp predicates which use the corresponding feature when examining rule by rule. Moreover, in such a way, the crisp boundaries expressed in each predicate for the numeric features involved are discharged. Thus, on the one hand, the same fuzzy predicate can be generated from two different instances of a specific form of crisp predicate appearing in two different rules. On the other hand, by working at rule level, even if, in more than one rule, many crisp predicates share a same numeric feature with different crisp boundaries, its universe of discourse is not further partitioned in order to consider the possible sub-partitions identified by the overlapping of different crisp intervals. So, any relationship that could exist between the rules in terms of shared features is not taken into account. Both these methodological choices, which can appear as a simplification and a loss of generality, are guided by the consideration that defining a justifiable, i.e. limited, number of terms for each variable is another determinant of interpretability [16]. Indeed, taking into account all the possible overlapping of different crisp predicates in terms of numeric intervals, by observing the whole *CRB*, in order to deeply partitioning a shared feature, would generate an overfitting of its universe of discourse, compromising the overall interpretability.

Furthermore, in the case of categorical features, the method generates a collection of as many terms for each linguistic variable as the crisp predicates occurring in the whole rule set which assign a different categorical value to the feature used.

For what concerns the predicted class, since it is also a categorical variable, the number of terms associated to the corresponding linguistic variable is assumed to be equal to the number of different values the predicted class can assume.

As a result, the fuzzification of the reduced rules generated a linguistic variable for every feature appearing in their antecedent parts, namely perimeter, texture, concave points and radius, each of them characterized by at most two partitions for the corresponding Universe of Discourse. Moreover, the output class, i.e. diagnosis, was also modeled as a linguistic variable assuming two specific values, namely malignant and benign. The resulting fuzzified rules are outlined in Figure 9, bottom), where linguistic variables and their terms are indicated with their first letter capitalized.

Concerning the generation of the other parameters pertaining the final FIS underpinning the DDSS, the terms *Low *and *High *generated after the fuzzification for the input variables were modeled with piece-wise linear membership functions, whereas the terms *Malignant *and *Benign *for the output variable were represented as singletons. The Singleton-type reasoning mechanism was used, where min and max operators were chosen as T-norm and S-norm functions, respectively. Moreover, the min operator and the center of gravity singleton methods were applied for implementing the implication and defuzzification functions, respectively. A not-chained Fuzzy Inference Engine was used by wrapping the implementation given within the JfuzzyLogic tool.

**Figure 10:**
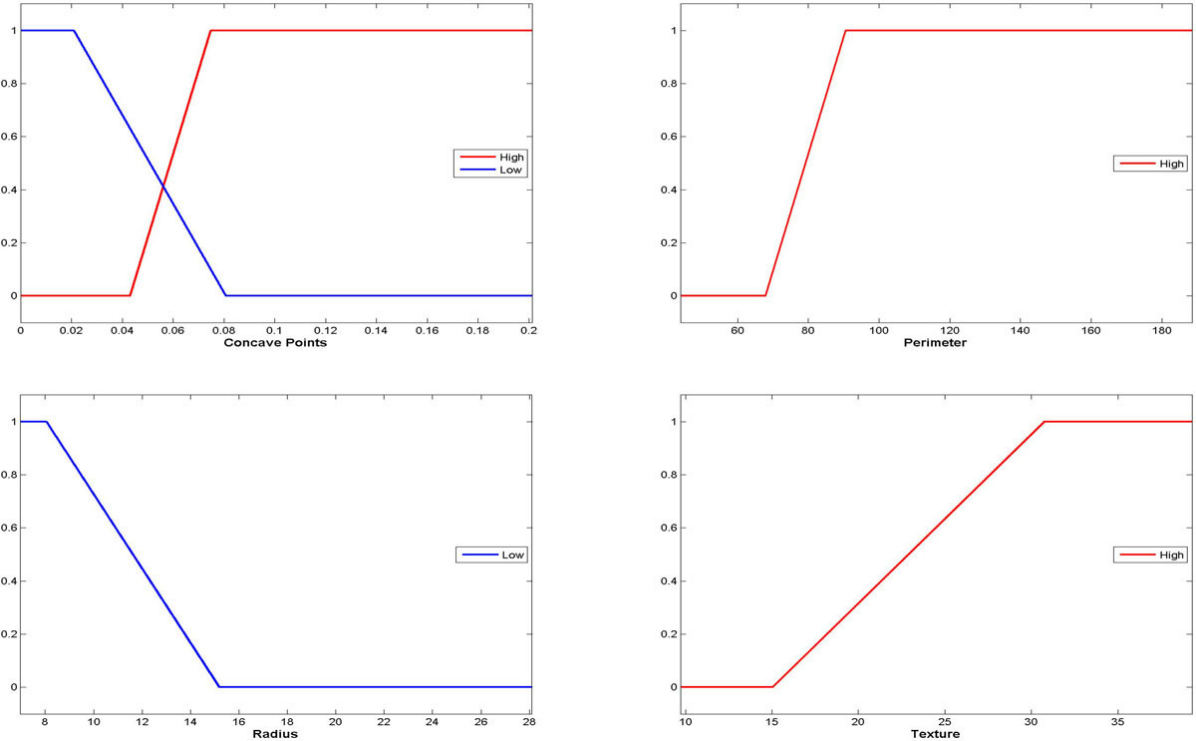
**The fuzzy partitions adapted for each linguistic variable**.

Finally, adaptation based on the DeltaJump algorithm was carried out for optimizing only the membership functions linked to the terms of the linguistic variables involved into the fuzzy rules obtained. Such an algorithm was wrapped on the top of the implementation provided by the JfuzzyLogic tool. Only a metric based on straight mean square error is used for the evaluation with respect to the classification rate, while interpretability [6] and confidence [4] are not considered yet. The fuzzy partitions achieved for each linguistic variable are outlined in Figure 10.

**Figure 11:**
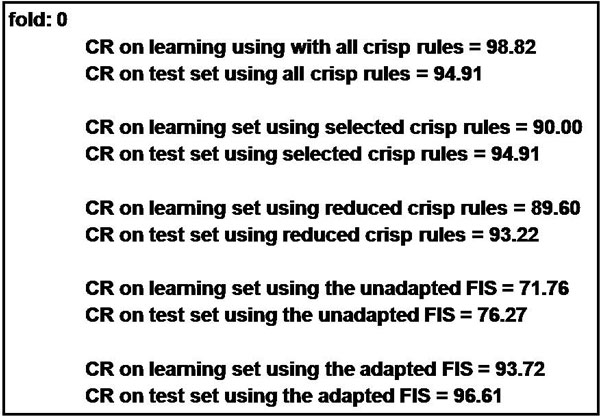
**The results achieved after the validation in terms of classification rate**.

These results were finally validated with respect to the classification rate measured for the DDSS instantiated on the first fold for the WBCD dataset. In more detail, they were calculated on both the training and learning sets, depending on the rules achieved in the different stages of the methodology, i.e. ranging from the crisp rules obtained at the end of rule extraction, to their selected and reduced version, until the fuzzified rules before and after their adaptation. The validation results are outlined in Figure 11.

**Figure 12:**
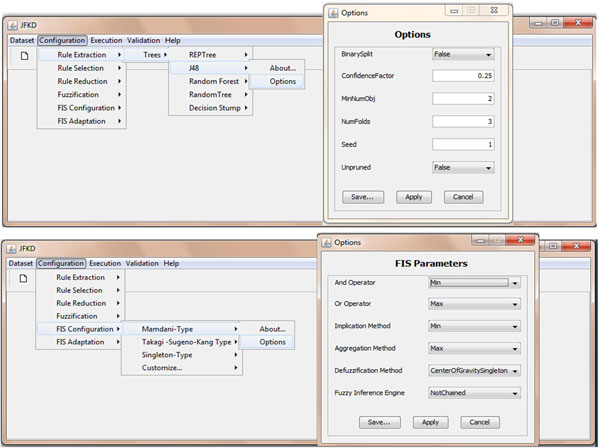
**The implemented system: how to configure each stage of the methodology**.

**Figure 13:**
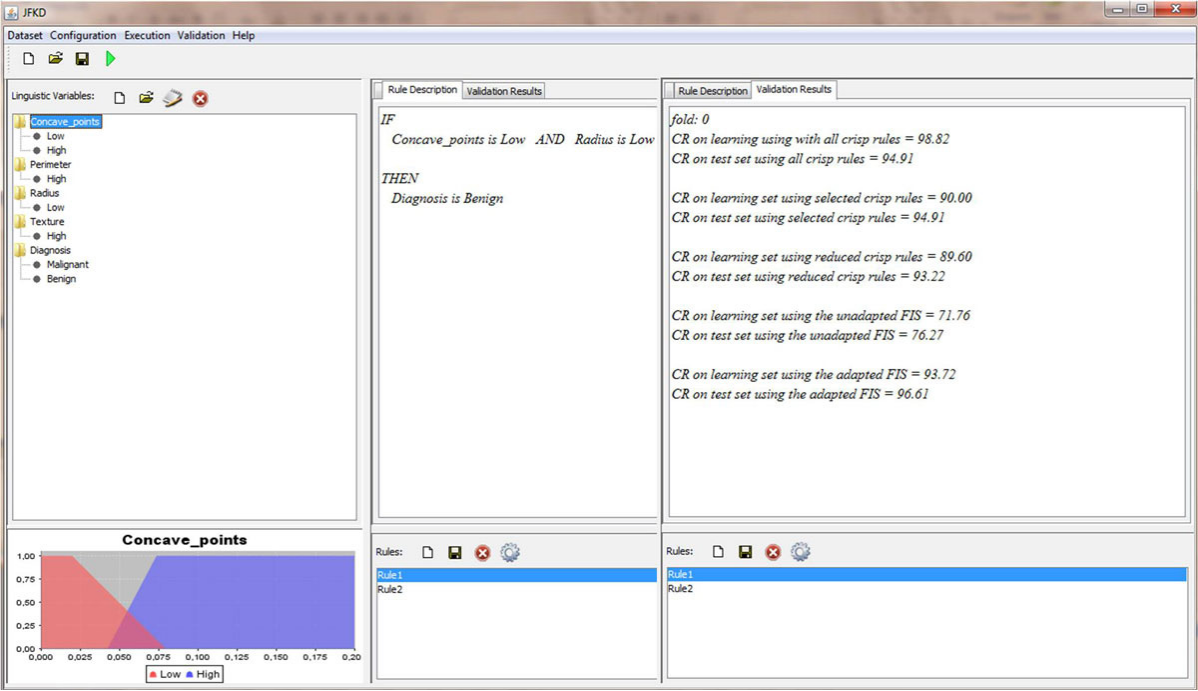
**The implemented system: the validation results with respect to a specific metric indicated**.

Figures 12 and 13 sketch the GUI implemented for facilitating the construction of a DDSS on the top of the proposed architecture: the user is asked to specify the dataset to be used, the algorithms or techniques to be adopted in all the stages of the methodology with all the parameters required for their configurations, and, finally, the validation method for evaluating the results with respect to a specific metric or index indicated (Figure 12).

Figure 13 reports the results achieved in terms of fuzzy rules extracted, linguistic variables and terms involved in the rules and, for each term, the corresponding membership function optimized. Moreover, such results are also enriched by means of the values assumed by the quantitative metrics used for the validation.

## Conclusions

Having in mind to extend the range of possible users of fuzzy-based DDSSs with extensive and easy-to-use facilities which could considerably reduce the level of knowledge and experience required to their design and realization, this work has first presented a formalization of a refined and assessed version of a six-step methodology to design and implement fuzzy-based DDSSs. Its strength relies on its generality and modularity since it supports the integration of alternative techniques in each of its stages. Stages are employed for: *(i) *the extraction of crisp rules, *(ii) *the selection of a significant partition from the whole rule set extracted, *(iii) *the reduction of the selected rule set, *(iv) *the creation of fuzzy rules, *(v) *the generation of the whole fuzzy inference system and *(vi) *its optimization.

Differently from other existing approaches [14], [15], the described methodology is extremely flexible and does not depend on the typology of fuzzy model to be defined, since it enables the design and realization of fuzzy-based DDSSs by taking into account many different and often conflicting requirements, such as the accuracy maximization or the complexity minimization. In more detail, it can not only integrate state-of-the-art rule-induction and rule-optimization methods, but also freely choose the structural and operational elements of the fuzzy model to be used, such as shape of membership functions or the t-norm and s-norm connectors as well as the implication and defuzzification operators. Moreover, methods that use shared fuzzy sets for the rule base [16] are appropriate within a small size work space with a good coverage. Otherwise, in case of a weak coverage the rule base completeness is not guaranteed and, when dealing with large systems, the number of combinations to manage is huge [16]. On the contrary, the proposed methodology is well adapted for large work spaces and generates more compact incomplete rules with only the most locally significant variables, defined successively with a partitioning strictly dependent on the rules where they are involved.

The presented methodology has been realized, according to a CBSD approach, in the form of a modular and portable architecture that has been carefully described from a software engineering perspective.

This architecture has been conceived to support the design of a fuzzy-based DDSS on increasing levels of abstraction, thereby partitioning the overall design problem into several sub-problems, where each single component at every layer can be implemented from scratch or customized by existing available solutions. Such a way, it can significantly reduce development effort and time-to-market, and improve maintainability, reliability and overall quality of final CDSS designed.

The development of this architecture has been last carried out by using the Java language since it contains several features that argue for it. It is widely distributed and has become one of the major programming languages. The development kit, including compiler and debugger, is freely available on a number of different computer platforms. The core libraries contain many functions which can be used directly and need to not be adopted from external libraries, which is not the case in C++ for instance. By exploiting Java features and diffusion in the user-community, the proposed architecture has several unique advantages, e.g. it reduces programming work. Thanks to the huge amount of available Java software, in fact, it is really easy creating new methods to be added to the Method Layer of the architecture without the effort of starting from scratch. Such a way, it is possible to exploit the richness of quickly incorporating new developments made by the active research community which is always working in emerging fields. Moreover, due to the use of a strict object-oriented approach for the its components, the architecture can be used on any machine with Java. Indeed, the concept of modularity of code is highly essential to increase the level of portability. As a result, any user can apply the architecture to implement a DDSS on his machine, independently of the operating system.

As a proof of concept, such an architecture has been used to instantiate a DDSS example aimed at accurately diagnosing breast masses starting from the widely used *Wisconsin Breast Cancer Dataset*. The results obtained in terms of classification rate proved the feasibility of the whole methodology implemented in terms of the architecture proposed.

For what concerns the on-going work, knowledge representation techniques such as ontology modeling are investigated to be exploited in order to better define from a semantic point of view the fuzzy variables and terms involved into the rules and improve the readability and understandability of the whole fuzzy-based DDSS. The choice of using Java as programming language will be able to facilitate this integration since the most representative tools in the context of knowledge engineering are implemented in Java and released as open source projects. Moreover, since DDSSs, however, typically have unequal classification error costs so that straight *CR *cannot be assumed as a careful measure of the goodness of a DDSS, in the future, also the confidence χ will be evaluated to be used for selecting a DDSS; in fact, a *good *DDSS should be highly confident with correctly classified examples while it should be doubtful with misclassified data points. In such a direction, also more sophisticated adaptation techniques able to optimize multi-objective cost functions will be integrated, so taking into account simultaneously *CR*, the confidence χ and the interpretability. The last important point for future work is to integrate the multi-threading and distributed computing to speed computations up during the definition and the adaptation of the fuzzy-based DDSS by using widely available multi-processors and multi-core hardware.

## Competing interests

The authors declare that they have no competing interests.

## Authors' contributions

All the authors contributed to the formalization of the methodology, the design of the architecture and the realization and testing of its implementation. All the authors cooperated to the writing of the manuscript, read and approved its final version.

## Declarations

The publication costs for this article were funded by the MERIT project.

This article has been published as part of *BMC Bioinformatics *Volume 14 Supplement 1, 2013: Computational Intelligence in Bioinformatics and Biostatistics: new trends from the CIBB conference series. The full contents of the supplement are available online at http://www.biomedcentral.com/bmcbioinformatics/supplements/14/S1.
